# Investigating the Pathogenesis of Severe Malaria: A Multidisciplinary and Cross-Geographical Approach

**DOI:** 10.4269/ajtmh.14-0841

**Published:** 2015-09-02

**Authors:** Samuel C. Wassmer, Terrie E. Taylor, Pradipsinh K. Rathod, Saroj K. Mishra, Sanjib Mohanty, Myriam Arevalo-Herrera, Manoj T. Duraisingh, Joseph D. Smith

**Affiliations:** Division of Parasitology, Department of Microbiology, New York University School of Medicine, New York, New York; Department of Pathology, Sydney Medical School, The University of Sydney, Sydney, Australia; Department of Osteopathic Medical Specialties, College of Osteopathic Medicine, Michigan State University, East Lansing, Michigan; Blantyre Malaria Project, University of Malawi College of Medicine, Blantyre, Malawi; Departments of Chemistry and Global Health, University of Washington, Seattle, Washington; Department of Internal Medicine, Ispat General Hospital, Orissa, India; Caucaseco Scientific Research Center, Cali, Colombia; Department of Immunology and Infectious Diseases, Harvard School of Public Health, Boston, Massachusetts; Seattle Biomedical Research Institute, Seattle, Washington; Department of Global Health, University of Washington, Seattle, Washington

## Abstract

More than a century after the discovery of *Plasmodium* spp. parasites, the pathogenesis of severe malaria is still not well understood. The majority of malaria cases are caused by *Plasmodium falciparum* and *Plasmodium vivax*, which differ in virulence, red blood cell tropism, cytoadhesion of infected erythrocytes, and dormant liver hypnozoite stages. Cerebral malaria coma is one of the most severe manifestations of *P. falciparum* infection. Insights into its complex pathophysiology are emerging through a combination of autopsy, neuroimaging, parasite binding, and endothelial characterizations. Nevertheless, important questions remain regarding why some patients develop life-threatening conditions while the majority of *P. falciparum*-infected individuals do not, and why clinical presentations differ between children and adults. For *P. vivax*, there is renewed recognition of severe malaria, but an understanding of the factors influencing disease severity is limited and remains an important research topic. Shedding light on the underlying disease mechanisms will be necessary to implement effective diagnostic tools for identifying and classifying severe malaria syndromes and developing new therapeutic approaches for severe disease. This review highlights progress and outstanding questions in severe malaria pathophysiology and summarizes key areas of pathogenesis research within the International Centers of Excellence for Malaria Research program.

## Introduction

Malaria is a major global infectious disease caused by parasitic protozoans of the genus *Plasmodium*. Of the five *Plasmodium* species that infect humans, *Plasmodium falciparum* and *Plasmodium vivax* cause the majority of cases, and *P. falciparum* is the most virulent and responsible for the majority of deaths.^1^ Despite recent reductions in the overall malaria case incidence, malaria remains a leading cause of morbidity and mortality in the developing world. In 2012, there were an estimated 207 million cases of malaria and over 600,000 deaths.^1^ The majority of malaria deaths (90%) occur in children in Africa, where falciparum malaria accounts for as many as one in six childhood deaths and is the biggest killer of African children between the ages of 1 and 4 years.^2^,^3^ Outside Africa, there are a variety of transmission settings where *P. falciparum*, *P. vivax*, or both are present. In lower transmission settings in South America, India, and southeast Asia, adult populations are at higher risk for severe malaria.

Malaria is a complex disease, and the spectrum of disease manifestations differs between children and adults.^4^ Symptoms can range from none, in individuals with asymptomatic parasitemia, to mild, in patients with undifferentiated fever, to severe, in patients with life-threatening anemia, metabolic acidosis, cerebral malaria (CM), and multiorgan system involvement.^5^ Only a small minority of infections, less than 1–2%, leads to severe malaria.^6^ Because pathogenetic mechanisms are complex and poorly understood, current treatment primarily relies on antimalarial drugs and supportive care. Here we focus on recent advancements in understanding the molecular pathogenesis of CM and the variable presentations between children and adults.

Several pathogenetic mechanisms have been proposed for CM including mechanical microvascular obstruction by sequestered infected erythrocytes (IEs),^7^ activation of immune cells and release of pro-inflammatory cytokines,^8^,^9^ endothelial dysfunction,^10^ dysregulation of coagulation pathways,^11^,^12^ blood–brain barrier (BBB) permeability,^13^ and brain swelling.^14^ Furthermore, autopsy studies have subdivided pediatric cases into two different groups based on histopathological patterns. The CM1 group has sequestration only, while CM2 group has sequestration plus vascular pathology (ring hemorrhages, fibrin-platelet thrombi, and monocytes).^15^,^16^ Ring hemorrhages and cerebral thrombosis are also described in a proportion of adult cases,^17^ but whether there is an equivalent CM1/CM2 dichotomy in adults is less clear. Recent findings implicate a specific subset of parasites that adhere to endothelial protein C receptor (EPCR) in severe childhood malaria.^18^ As EPCR plays a key role in regulating coagulation and endothelial cytoprotective and barrier properties,^19^ this raises the possibility there may be linkages between IE cytoadhesion and microvascular complications in CM.^20^ However, the precise molecular processes that account for the pathophysiological differences between CM1, CM2, and adult CM are poorly understood. Elucidating key pathogenetic mechanisms in CM and severe malaria may suggest new treatment options to improve patient outcomes.

Unlike *P. falciparum*, *P. vivax* rarely causes severe disease in healthy travelers and is a less deadly parasite.^21^ Factors that may contribute to the lower virulence are that *P. vivax* only infects reticulocytes and the absence of the cytoadhesion protein family responsible for sequestration in *P. falciparum* infections.^21^,^22^ These differences limit the blood-stage parasite burden and spectrum of cytoadhesion-based complications. Another distinction is that *P. vivax* has dormant liver hypnozoite stages, which can reactivate and lead to blood-stage relapses. Relapses contribute to vivax morbidity, but the mechanisms leading to severe vivax disease remain to be elucidated. This review covers recent findings on the pathological pathways in pediatric and adult CM, as well as severe malaria cases in low-transmission settings in South America and India because of *P. vivax* infections, highlighting progress and outstanding questions in severe malaria pathophysiology in the context of the pathogenesis research activities within the International Centers of Excellence for Malaria Research (ICEMR) program.

## Severe Falciparum Malaria in Children and Adults

**Figure 1.**
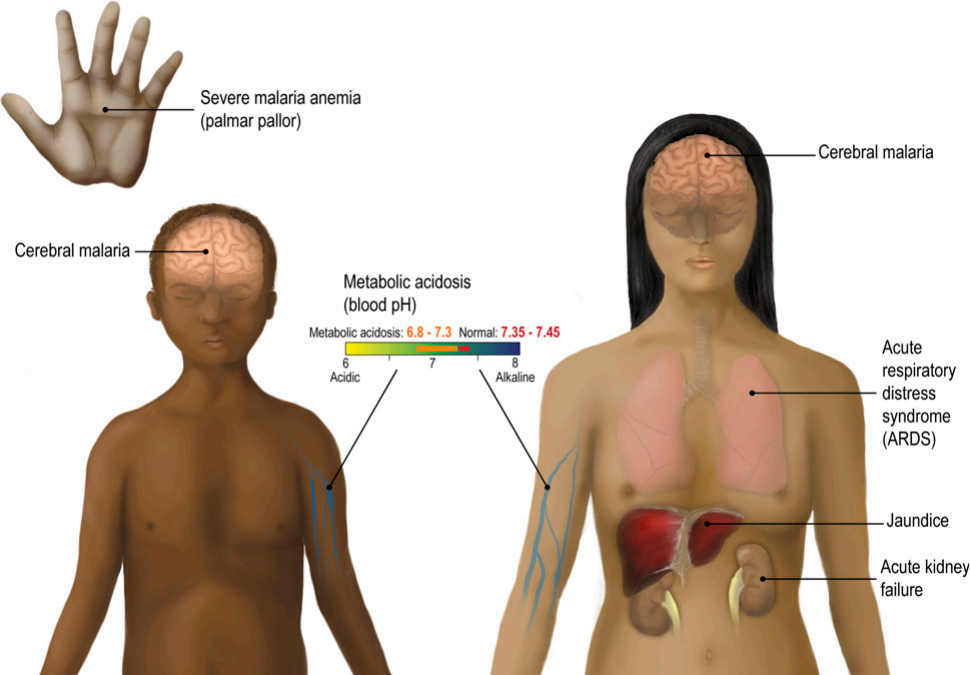
The major clinical complications associated with adult and pediatric severe malaria. Clinically severe malaria is a multisystem disorder that can affect different organs and differs in presentation between children and adults. The major clinical complications in children are cerebral malaria, severe malaria anemia, and metabolic acidosis. In adults, cerebral malaria is frequently accompanied by multiorgan system complications, including metabolic acidosis, acute kidney failure, jaundice, and acute respiratory distress (ARDS).

The clinical presentations of severe falciparum malaria differ between children and adults.^5^ In particular, adults have a higher mortality rate and more multiorgan system involvement than children. A recent large multicenter comparison of artesunate versus quinine in the treatment of severe malaria in adults and children reported adult and pediatric mortality rates of 18.5%^23^ and 9.7%, respectively.^24^ The major organs affected in adult severe malaria are brain (CM), lungs (acute respiratory distress syndrome [ARDS]), liver (jaundice), and kidneys (acute renal failure) (Figure 1

). Although the overall mortality of adult CM is about 15–20%, the risk of death depends on associated vital organ dysfunction and is increased 3-fold in the presence of acidosis and renal failure.^25^ In children, the three major disease complications are CM, severe anemia, and acidosis, but ARDS and renal failure are rare (Figure 1).^26^ Although the three disease syndromes can occur singly or as overlapping syndromes, severe malaria anemia commonly affects younger children, and CM and metabolic acidosis are more commonly found in slightly older children.^27^ CM and metabolic acidosis are each associated with high mortality rates in children (12% and 14%, respectively), and the presence of both increases the risk of death.^28^ The severity of disease may be exacerbated by both higher parasite burdens and the tissue-specific patterns of IE sequestration. Thus, there is significant research effort to understand factors that contribute to parasite blood-stage multiplication potential and cerebral homing of IEs.

Although severe malaria predominantly affects African children in high-transmission settings and adults in lower transmission settings, the same differences in disease complications and mortality were observed between adults and children in Rourkela, India.^29^ Collectively, these findings suggest there are different pathophysiological disease mechanisms in children and adults, but the molecular mechanisms underlying these differences are not fully understood. The different clinical symptoms could result from differences in host malaria immune status, since malaria transmission intensity is much higher in Africa than other regions where adults experience severe malaria. Alternatively, they could potentially relate to different parasite binding types, human polymorphisms, or age-dependent changes in the vascular system response to falciparum-induced inflammation.

### *P. falciparum* and cerebral malaria: a histopathological and ultrastructural perspective.

A major pathological feature of *P. falciparum* malaria is that the mature stage IEs sequester from blood circulation by binding to the endothelial lining of blood vessels. Histopathological studies of fatal malaria had largely focused on adults in hypoendemic areas^30^,^31^ and soldiers in military theatres,^17^ until recently, when a group based in Malawi undertook a case–control study, comparing the gross and microscopic pathology in children dying with clinically defined CM to the pathology in malaria-infected children with non-malarial causes of death.^16^

**Figure 2.**
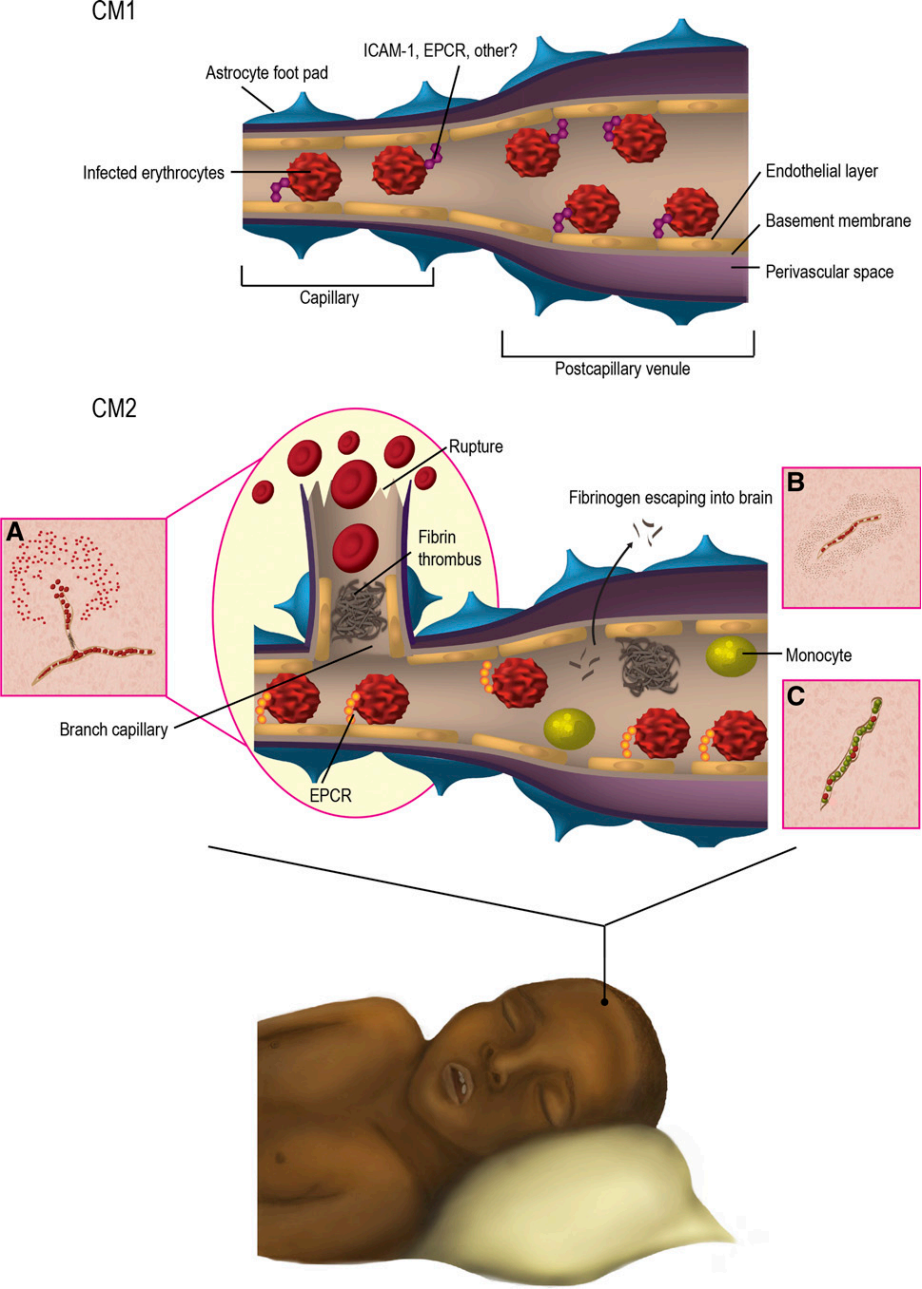
Schematic representation of the pathological differences between cerebral malaria CM1 and CM2. Autopsy studies in children have divided CM cases into two groups based on histological features,^16^ CM1 cases have infected erythrocyte sequestration in the cerebral microvasculature and no associated vascular pathology. CM2 cases are defined by cerebral sequestration plus intra- and perivascular pathology, including ring hemorrhages, fibrin-platelet thrombi, and intravascular monocytes. In the CM2 group, infected erythrocyte (IE) sequestration is frequently associated with fibrin-platelet thrombi in both capillaries and postcapillary venules. Insets provide examples of pathological features observed in CM2 cases described in Dorovini-Zis and others.^15^ Inset (**A**) shows a small branching capillary in which the upstream region is filled with sequestered IEs and one of the branches is occluded by a thrombus. This event is associated with a ring hemorrhage in which the microvessel is partially denuded of endothelial cells and is surrounded by a zone of necrosis and a ring of uninfected red blood cells in the white matter. Inset (**B**) shows a small vessel packed with sequestered IEs and surrounded by extravasated fibrinogen indicating increased permeability of the blood–brain barrier. Inset (**C**) shows a micovessel filled with monocytes containing phagocytosed hemozoin pigment. Intravascular pigmented monocytes are found adherent to the microvessel wall, but do not transverse across the blood–brain barrier. The molecular mechanisms driving the CM1 and CM2 pathophysiology are incompletely understood. Intercellular adhesion molecule 1 (ICAM-1) and endothelial protein C receptor (EPCR) are candidate brain endothelial receptors,^18^,^31^ but it is not known if the same parasite adhesion types are associated with CM1, CM2, and adult CM (not pictured). Recent studies reported that binding of IE to EPCR was associated with the development of severe malaria^18^ and that decreased EPCR staining on endothelial cells and increased fibrin deposition occurred at the site of IE adhesion in cerebral microvessels during fatal pediatric CM.^20^ This association suggests there may be causal links between cytoadhesion and microvascular pathophysiology. However, fibrin deposition is not found in CM1 and is less prominent in adult CM, highlighting gaps in our understanding of CM pathophysiology.

A striking finding was that ~25% of children who met the standard clinical case definition of CM during life (*P. falciparum* parasitemia, Blantyre Coma Score ≤ 2, no other obvious cause of coma)^32^ had no evidence of the pathological hallmark of CM, the cerebral sequestration of IE. All of these children had a non-malarial cause of death identified at autopsy.^16^ This finding highlights the difficulty of assigning the true cause of coma in children in geographic regions with high rates of apparently asymptomatic malaria infections and emphasizes the need for better CM diagnostics to guide treatment decisions. Among those who did have evidence of cerebral sequestration of IEs (“true CM”), two distinct pathological patterns were noted, CM1 and CM2 (Figure 2

). Approximately one-third of the true CM patients had histologic evidence of sequestration only (CM1); the other two-thirds had evidence of intra- and perivascular pathology (fibrin thrombi, “ring” hemorrhages involving uninfected red cells, and intravascular accumulation of white blood cells).^16^ Although fibrin and intravascular monocytes are less prominent features in adult CM autopsy studies, ring hemorrhages are found in approximately 30–50% of adult cases (Table 1 ). Furthermore, in the classic histopathological study of Spitz^17^ on World War II U.S. military soldiers who died of acute falciparum malaria, thromboses and ring hemorrhages were commonly observed together, leading Spitz to speculate that ring hemorrhages were caused by thrombosis. Thus, although the CM2 pattern is not commonly described in adult cases (Table 1),^42^ it is possible that thrombotic lesions may play a role in some adult CM cases. Overall, the variability of pathological findings indicates that CM is not a histologically uniform syndrome and suggests there may be different pathophysiological mechanisms in CM1 and CM2, and potentially between children and adults.

From histopathological studies, activation of endothelial cells^31^ and breakdown of the BBB are evident.^15^,^39^,^43^ Parasites can stimulate intracellular signaling events in endothelial cells whether through direct adhesion to receptors such as CD36 or intercellular adhesion molecule 1 (ICAM-1)^44^,^45^ or release of soluble factors.^45^,^46^ This affects cerebral endothelial cell structure and function, which in turn may mediate changes in the BBB function in CM,^13^ but the parenchyma of the brain is rarely involved. Nevertheless, in both children and adults, neuropathology has been associated with ring hemorrhages (Table 1), and if sufficient time has passed after an insult Durck's granulomas can develop at sites of ring hemorrhage. Durck's granulomas are occasionally seen in adults,^17^ but are extremely rare in children.^33^ More commonly, areas of demyelination are associated with IE sequestration in children^15^ and areas of axonal injury/activation or myelin loss have also been described in adult CM.^40^ However, the overall pattern of injury varies between children and adults. The molecular mechanisms underlying these differences still remain to be elucidated.

Increased brain volume was evident in all true CM cases in the Malawi series,^33^,^42^ but is not universal in adults.^47^,^48^ Several possible mechanisms may contribute to increased brain swelling, including 1) increased blood volume resulting from microvascular congestion generated by sequestered IEs and decreased venous outflow; 2) cytotoxic edema (BBB remains intact but accumulation of intracellular fluid due to altered metabolism or movement of water into brain cells occurs); 3) vasogenic edema (BBB is disrupted); and/or 4) increased cerebral blood flow volume in response to fever, anemia, and seizures. Understanding the relative contribution of these potential mechanisms to brain swelling may suggest treatment strategies.

### Imaging approaches to investigate disease pathogenic mechanisms.

As illuminating as autopsy studies have been, they are inherently limited by the necessity of only studying patients who have died, and by only studying them at one point in the process, the time of death. Imaging modalities that could be used during life, which could be repeated to capture a process, would be helpful in studies of malaria pathogenesis.

#### Orthogonal polarization spectral imaging.

Clear images of microcirculatory blood flow in mucosal surfaces (sublingual, rectal) obtained via orthogonal polarization spectral imaging allow for “real-time” visualization of microvascular obstruction related to sequestered IEs.^49^ This approach has revealed significant disturbances in microvascular blood flow that were variable between adjacent microvessels and increased in proportion to disease severity. These abnormalities disappeared after patient recovery, highlighting an important role for reduced microcirculatory blood flow in severe malaria. However, because the expression of surface receptors varies between organs,^50^ what is seen in accessible areas may not reflect what is happening in the brain.

#### Ocular funduscopy.

The eye and the brain have similar embryologic origins, and the microvasculatures of the two organ systems share important features."bibr">51 In addition, the optic fundus can be readily observed and studied during life in patients with severe malaria. In conjunction with the Malawi autopsy study, ophthalmologists described a unique malarial retinopathy consisting of white-centered hemorrhages, vessel color changes, and peri- and extramacular whitening.^52^ At least one of these findings was present in all cases of true CM (i.e., patients with evidence of cerebral sequestration of IEs at autopsy), and although recognition of the retinopathy requires a trained observer with relatively expensive equipment (direct and indirect ophthalmoscopes), it has created the opportunity, exploited by the ICEMR program, to use a more specific clinical case definition of CM. Retinal hemorrhages correlate, numerically, with the ring hemorrhages seen in fatal cases of pediatric CM.^53^ Vessel color changes reflect the presence of sequestered, parasitized, and de-hemoglobinized red cells,^54^ while the whitening represents areas of impaired perfusion.^55^

Ophthalmologic observations on adults with severe malaria are relatively sparse, but they are consistent with the reported pediatric findings in that approximately one-third of adults meeting the standard clinical case definition of CM have no evidence of malarial retinopathy.^56^ Retinal hemorrhages are commonly observed,^57^ but vessel color changes, seen in ~32% of children with CM,^58^ are only rarely seen in adults.^56^ The severity of malaria retinopathy is strongly associated with malaria mortality in both adults and children.^51^,^56^

#### Neuroimaging.

Neuroimaging neatly addresses the two primary deficiencies of the autopsy approach: survivors can be imaged and serial studies can be carried out throughout the course of the acute illness. However, the worldwide distribution of sophisticated radiological capacity does not include malaria-endemic areas, so most descriptions of neuroimaging findings in malaria patients have been single case reports from patients hospitalized in more developed countries.^14^

Computed tomography scan technology is relatively uncomplicated and affordable, and the process itself is quick. This approach was the first used to illuminate disease pathogenesis in malaria patients, and highlighted the importance of increased brain volume.^59^–^61^ Most of these studies were done before the importance of malarial retinopathy was recognized, though the possibility of classification errors complicates interpretation of these findings.

Individual case reports of magnetic resonance imaging (MRI) findings in patients with CM (as reviewed in reference 59) have described a variety of findings, all of which have been corroborated by larger, systematic studies in Thai adults^62^ and Malawian children.^63^ Increased brain volume is strongly associated with a fatal outcome in children.^64^ Cortical involvement (often restricted to specific lobes), and changes in the periventricular white matter, the corpus callosum, and the thalami are common in children with retinopathy-positive CM.

Both of the larger studies were limited by the strength of the magnet (0.2 tesla [T] in Thailand, 0.35 T in Malawi). A collaborative effort between two independent ICEMR projects (Table 2 ) will address this problem while simultaneously addressing disparities between the clinical manifestations of severe disease in adults and children. The joint effort is currently being carried out between two hospitals, one located in Malawi and one in India, both of which have MRI facilities. Adults and pediatric patients with severe malaria in India (retinopathy-positive CM, with and without other organ system involvement) will undergo MRI on a 1.5 T machine, and their findings will be compared with those in retinopathy-positive CM pediatric patients in Malawi. The clinical protocol has been standardized between the two field sites, and four MRI sequences will be common to both projects, as their magnet strengths are different. To ensure the accurate interpretation and comparison of MRI findings in these sequences, all the images will be scored and shared between the radiologists, via a web-based platform to enhance standardization.^65^ This study will permit, for the first time, the clinical characterization of pediatric and adult CM by neuroimaging and a precise comparison of carefully clinically defined cohorts of CM patients of different ages and from different continents. Such extensive MRI techniques have never been applied systematically to patients with acute malaria and represent a promising approach to investigating the relationship between brain swelling and the onset of CM.

### Vascular activation/dysfunction and coagulation pathways in severe malaria.

The brain swelling observed during CM both in Indian adults and Malawian children might be the consequence of disruption of the BBB associated with the pathogenetic processes of CM, resulting in vasogenic edema. This hypothesis is currently being investigated as part of a Malawi–India inter-ICEMR initiative (Table 2) and is in line with the emergence of the endothelial cell as a central player in the pathophysiology of the neurologic syndrome. Although its involvement as a substrate for IE sequestration in the brain was identified very early on,^66^ results published over the past decade have highlighted the complex role of cerebral endothelial cells in the development of CM. One of the main goals of the India ICEMR is to investigate parameters inherent in the host endothelium that may result in an increased susceptibility to severe malaria in Indian adults infected with *P. falciparum*, an axis of research that is divided into three main approaches.

#### Variations and heritability of the host endothelial responsiveness to tumor necrosis factor alpha.

**Figure 3.**
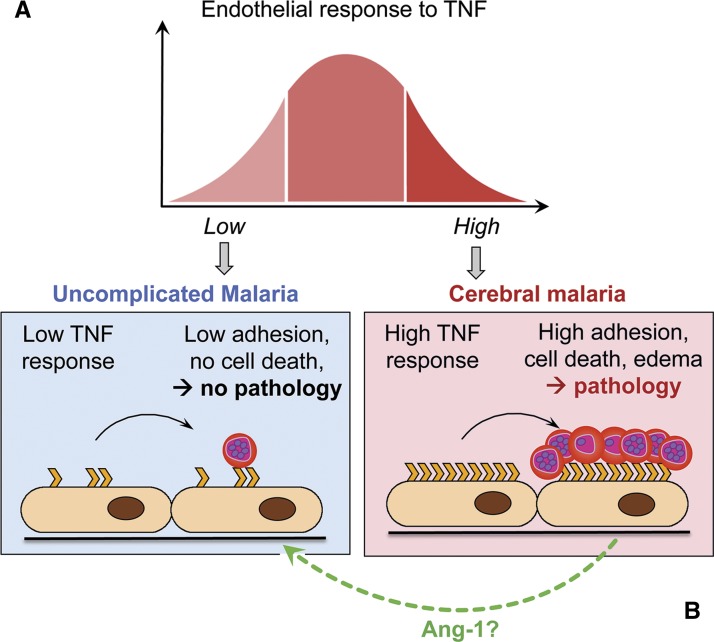
Proposed influence of the host endothelial responsiveness to tumor necrosis factor (TNF) on the severity of malaria infection. Low endothelial TNF responders are less prone to upregulate receptors involved in the sequestration of infected erythrocyte (IE) and platelets than high responders. This leads to a minimal adhesion of IE and host cells and a lower pro-apoptotic signal for the endothelial cells, which might account for the absence of pathology. High responders, however, are over-activated in the presence of TNF, leading to high adhesion of IE and a strong pro-apoptotic signal, possibly resulting in the breakdown of the blood–brain barrier and, ultimately, to vasogenic edema (**A**). Potential clinical benefits offered by angiopoietin (Ang)-1 as a quiescence agent for high TNF-responding endothelial cells during cerebral malaria (CM) (**B**).

A central component of CM pathophysiology is the activation of microvascular endothelial cells, resulting from both the cytoadherence of IE to their surface and the pro-inflammatory effects of local and systemically released cytokines.^67^ The consequences of this endothelial inflammation are numerous and include the upregulation of endothelial receptors for enhancing IE and platelet sequestration; the further release of cytokines and chemokines and the trigger of a tumor necrosis factor (TNF)–dependent pro-apoptotic pathway (as reviewed in reference 68). We hypothesized that variation in the responsiveness of endothelial cells to TNF in different individuals could be a factor affecting the severity of disease in patients infected with *P. falciparum* (Figure 3A

). Indeed, endothelial cells derived from CM and uncomplicated (UM) children patients from Malawi were shown to display significantly different ex vivo responsiveness to TNF. When compared with UM, CM-derived endothelial cells express significantly higher levels of parasite and platelet receptors, produce more endothelial microparticles, release more pro-inflammatory cytokine, and are more prone to undergo apoptosis on stimulation with TNF.^69^ On the basis of these results, it was hypothesized that genetic variations within promoter, intron, or exon sequences of endothelial inflammatory genes may, in part, determine the clinical course in CM patients, as has been described in sepsis.^70^

Using a large number of freshly isolated microvascular endothelial cells from adult patients admitted to Ispat General Hospital in Rourkela, we are planning to compare the response to TNF between CM and UM patients from India and investigate the different factors, extrinsic or intrinsic, leading to the interindividual differential activation of the endothelium between the two patient categories. The comparative analysis of the variation in transcripts between the two high and low TNF-responding groups of endothelial cells will give us insights into the pathways involved in the acute activation observed in CM patients, and will be compared with the results obtained in Malawian children. This project is carried out not only with a view to understanding the molecular basis of disease but also to identifying patients at risk by analyzing specific single nucleotide polymorphisms associated with high and low responders. It will also assess if there are age-specific differences in endothelial responsiveness. Understanding the mechanistic basis of vascular dysfunction in severe malaria may suggest new treatment options.

#### Reversibility of the systematic endothelial activation in CM patients.

The presence of TNF as a trigger of inflammation in malaria led to the assessment of a TNF-blocking approach in CM. Although in vitro treatments produced favorable results, anti-TNF clinical trials failed to reduce mortality in these patients.^71^,^72^ The use of a targeted compound blocking the downstream endothelial activation signaling cascade resulted in a reduction of endothelial inflammation in vitro.^73^ However, this effect was only observed when the compound was administered simultaneously with the cytokine, which would be effectively impossible in vivo. Since most of the patients admitted to the ward have already high levels of TNF, an acute therapy might work by dampening the existing endothelial inflammatory response in CM patients. Angiopoietin (Ang)-1 has recently become a topic of increasing interest in endothelial cell quiescence and survival,^74^ and plasma Ang-2/Ang-1 ratio has been shown not only to be crucial for the endothelial activation but also to discriminate UM and CM. Indeed, high levels of Ang-2 are associated with mortality in patients with CM, whereas high levels of Ang-1 are associated with UM (as reviewed in reference 75). Since the use of Ang-1 offers clinical benefits as a quiescence agent for endothelial cells in an elegant model of sepsis,^76^ it is conceivable that restoring the Ang-2/Ang-1 balance in favor of Ang-1 would block and potentially reverse the ongoing inflammatory processes in CM patients at the time of admission (Figure 3B).

The potential clinical benefits of Ang-1 are currently being evaluated as part of the ongoing project on primary endothelial cells at Ispat General Hospital. Using the endothelial cell banks isolated from CM patients, the effects of Ang-1 on TNF-stimulated endothelium will be measured, with a view to develop new adjunct therapies and improve disease outcome in CM.

#### The role of EPCR in adult CM.

Recent studies reported that binding of IE to EPCR was associated with the development of severe malaria^18^ and that decreased EPCR staining on endothelial cells and increased fibrin deposition occurred at the site of IE adhesion in cerebral microvessels during fatal pediatric CM.^20^ A causal relationship between cytoadhesion and coagulopathy was therefore suggested for the first time, and the pivotal role of EPCR in the organ specificity of the syndrome was proposed.^77^,^78^ One of the major aims of the India ICEMRs is to further investigate the role of EPCR in the development of CM in Indian adults, as fibrin deposition is a far less prominent pathological feature in southeast Asian adults than African children who succumb to CM (Table 1).^20^,^30^ Since endothelial cell cultured from subcutaneous fat resemble cerebral vascular endothelial cell and represent a useful ex vivo model for examining brain endothelial alteration in the context of CM,^69^ this approach is being carried out by performing phenotypical analyses of primary subcutaneous endothelial cells isolated from patients admitted at Ispat General Hospital, followed by targeted gene expression profiling (RNA and miRNA) and genetic analyses of genes selected for their relevance in the protein C pathway. The results will 1) contribute to a better understanding of the pathogenic mechanisms for childhood and adult disease, 2) assess the overall importance of EPCR in mediating the cytoprotective effects of activated protein C (APC) in the brain, and 3) evaluate new avenues of translational research. A collaborative protocol is currently being developed between the India ICEMR and the clinical team to extend these analyses to endothelial cells isolated from postmortem brain biopsies samples of fatal CM.

### Parasite biomass and severe malaria.

It is difficult to measure the total parasite biomass of *P. falciparum* (circulating and sequestered) from blood sampling because of the “hidden” sequestered component. To overcome this challenge, a new approach has been introduced by Dondorp and others^79^ in which the plasma concentration of a soluble parasite molecule serves as a surrogate for the total parasite biomass. *Plasmodium falciparum* histidine-rich protein-2 (HRP-2) is a water-soluble protein produced throughout the parasite life cycle and released largely (but not exclusively) at the time of schizont rupture.^79^,^80^ It has a long half-life and persists in the plasma for up to 21 days, even after successful treatment^81^; HRP-2 detection (present/absent) is the basis of many rapid diagnostic tests, but quantitative measures of HRP-2 can discriminate between retinopathy-positive and retinopathy-negative CM,^82^ can predict which children with uncomplicated malaria are more likely to deteriorate,^83^ and can distinguish between patients with complicated malaria, mild malaria, asymptomatic parasitemia, and non-malarial fevers.^84^ A model, based on plasma half-life of HRP-2 in vivo¸ production rates of HRP-2 in vitro, and parasite multiplication rates suggests that HRP-2 concentrations reflect total body parasite burden (sequestered and circulating parasites).^79^ In general, the associations between HRP-2 concentration and disease severity support the hypothesis that parasite biomass is a major determinant of malaria pathogenesis. However, a recent longitudinal birth cohort study of Tanzanian children followed from birth to 2–4 years of age indicated that while parasite burden was higher on average in severe malaria episodes, high parasite burden was insufficient to cause severe disease.^85^ Thus, high parasite burden appears to be an important determinant in severe malaria, but other factors may act in concert to precipitate severe malaria episodes.

### Parasite invasion pathways and malaria severity.

Higher parasite biomass is a risk factor for severe malaria and may be driving increased systematic inflammation, endothelial activation markers, and metabolic acidosis by microvascular obstruction. The circumstances leading to higher parasite burdens in severe malaria are likely multifactorial and incompletely understood. However, potential parasite factors are red blood cell (RBC) invasion efficiency and the cytoadhesion efficiency of infected RBCs. Mathematical modeling approaches suggest that invasion efficiency can be a significant driver of peak parasite density during an infection and concomitant pathogenesis.^86^ Rodent malaria parasites can shift from a nonlethal to a lethal form following a change in preference from reticulocytes to older normocytes resulting in huge increases in parasite biomass and pathology.^87^ In humans, there is evidence that the efficiency of the invasion process can be a virulence determinant in *P. falciparum* parasites.^88^ Clearly, this can be influenced by genetic polymorphisms within both the host and the parasite, as well as acquired immunity. In addition, the ability of parasites to invade RBCs using alternative receptors, known as invasion pathways, can facilitate immune evasion and persistence of malaria infections^89^ and ultimately contribute to malaria pathogenesis. Anemia may result from chronic low-burden infections.^90^

Invasion potential has been measured in two ways: by parasite multiplication rate and by selectivity of RBCs. Both have been shown to be strongly associated with the severity of *P. falciparum* malaria in one population in southeast Asia,^88^ suggesting the existence of parasite molecular factors that mediate pathogenesis through increased proliferation. However, a similar study was carried out with parasite isolates from Africa and no association was found between invasion efficiency, selectivity, and disease severity.^91^ It is not clear whether this is due to regional differences in parasites or in host factors, such as the level of acquired antimalarial immunity.

Previous work carried out in several varied geographical areas have shown that natural *P. falciparum* isolates are capable of using multiple ligand–receptor invasion pathways, and exhibit variation in pathway usage, suggesting mechanisms by which invasion efficiency could be altered via parasite-based mechanisms. These studies have shown that both sialic acid–dependent and sialic acid–independent invasion pathways are commonly used by parasites collected directly from infected humans, and a few isolates have been shown to be able to switch between the use of sialic acid–dependent and sialic acid–independent pathways. Switching of one isolate was associated with reduced invasion efficiency.^89^

With the genome sequenced, *Plasmodium* parasites have been found to possess a diverse number of ligands for invasion. Two superfamilies of invasion ligands, the reticulocyte-binding-protein-like (RBL) and the erythrocyte-binding-protein-like (EBL) have been identified.^92^ Much data from studies with *P. falciparum* suggests that each parasite ligand has a single cognate receptor, defining alternative invasion pathways and that there is a hierarchy of different ligand–receptor interactions. Further, variation can exist at the level of sequence and expression changes for these invasion ligands, suggesting a molecular basis for switching between the use of different invasion pathways, either for immune evasion, to change the parasite multiplication rate, and/or RBC selectivity. To better understand the molecular mechanisms driving higher parasite burdens in severe malaria, an ICEMR group in India is addressing the interplay between parasite invasion efficiency and IE cytoadhesion phenotypes in disease severity.

### Parasite adhesion and severe malaria.

As described above, cytoadhesion of IEs is a major virulence determinant for CM complications. Furthermore, high parasite burdens and the massive sequestration of IEs in different tissue beds and resulting microvascular obstruction may lead to metabolic acidosis.^4^ The majority of falciparum infections are not severe, which suggests that the parasite is relatively well adapted to sequester in microvessels without killing the host. Cytoadhesion of IEs is predominantly mediated through the *var* gene/*P. falciparum* erythrocyte membrane protein 1 (PfEMP1) family of adhesion proteins.^93^–^95^ PfEMP1 proteins are anchored at parasite-induced, knob-like protrusions on the erythrocyte membrane,^93^ exposing them to host antibodies. Clonal antigenic variation of *var* genes enables *P. falciparum* to evade antibody destruction and to bind to different host receptors.^96^ Each parasite encodes approximately 60 different *var* copies^97^ with limited overlap of *var* gene repertoires between parasite haplotypes.^98^ The vast intra- and interstrain diversity in PfEMP1 repertoires enables parasites to establish chronic infections and repeatedly infect hosts with different parasite genotypes. A fundamental question for pathogenesis is whether specific PfEMP1 and host-receptor interactions have a causal role in severe malaria.

Despite extensive sequence diversity, the majority of *var* genes can be classified into three main subfamilies (A, B, and C) on the basis of upstream gene sequence and chromosomal location.^99^ Interstrain sequence comparisons have also identified three unusual strain-transcendent *var* genes (*var1csa*, *var2csa*, type 3 *var*).^100^–^102^ Each PfEMP1 protein encodes multiple adhesion domains called Duffy binding-like (DBL) and cysteine-rich interdomain region domains.^95^ PfEMP1 adhesion domains are classified into different types (α, β, γ, δ, etc.) and subtypes based on sequence similarity.^101^,^103^ Using adhesion domain classification, interstrain sequence comparisons have revealed a small number of tandem domain arrangements of 2–4 domains, called domain cassettes (DC), which are unusually conserved between parasite genotypes.^101^

The prototypical example of a specific PfEMP1 and disease is malaria in pregnancy. In this case, the strain-transcendent VAR2CSA mediates placental binding.^104^,^105^ It has been more challenging to determine if a specific PfEMP1 subset is associated with CM because of the difficulty of studying the brain. Analysis of *var* gene expression in patients has suggested that most infections contain a heterogeneous population of parasites expressing a mixture of A, B, or C *var* genes. In hosts with limited malaria immunity and severe pediatric malaria, the ratio of PfEMP1 variants appears to be skewed toward higher group A expression.^106^–^108^ These findings suggest that group A PfEMP1 encode adhesion traits that facilitate parasite multiplication in malaria naive hosts and may include binding properties that predispose to severe malaria. As individuals acquire anti-PfEMP1 antibodies through repeated infections, the proportion of group B and C variants appear to increase.^108^,^109^ However, even in pregnant African women who have acquired considerable antimalarial immunity, there was high *var2csa* expression from parasites recovered from placenta, but mixed *var2csa* and A, B, C *var* expression from parasites circulating in the blood.^110^ Thus, the parasite strategy of having a heterogeneous population appears to persist even after individuals have acquired substantial antimalarial immunity.^106^–^108^

More recently, it was shown that parasites expressing PfEMP1 proteins encoding DC8 or DC13 are strongly selected on human brain microvascular endothelial cells in vitro^111^,^112^ and are highly expressed in children with severe malaria or CM.^113^ The DC8 is found in an unusual chimeric gene between groups B and A and the DC13 is restricted to group A variants. Both DC8 and DC13 proteins, as well as a subset of other group A variants, were found to encode a novel binding property for EPCR,^18^ the receptor for APC. As the APC–EPCR pathway plays a key role in regulating blood coagulation and endothelial barrier properties,^19^,^114^ this has raised the possibility that there may be a linkage between IE binding and CM pathogenesis. However, given the different clinical presentation and autopsy findings in children and adults (Table 1),^5^ an important question is whether different PfEMP1 variants are associated with CM1, CM2, and adult CM.

As discussed above, one possibility is that host polymorphisms or age-specific differences in endothelial responses may contribute to pathophysiological differences. Alternatively, different parasite binding variants may be associated with CM1, CM2, and adult CM. For instance, ICAM1 has also been proposed to be a cerebral sequestration receptor.^31^ Therefore, one possibility is that ICAM1^+^, EPCR^−^ binding variants play a more predominant role in CM1 where fibrin-platelet clots and ring hemorrhages are absent, whereas EPCR^+^ binding variants are predominant in CM2 (Figure 2). To evaluate if parasite binding phenotype influences disease pathogenesis, more information is needed on the binding specificity of DC8, DC13, and other group A–expressing parasites for ICAM1 and EPCR.^111^,^112^ In addition, multiple domains in DC8 PfEMP1 bind to brain endothelial cells.^115^ Therefore, this analysis should include defining the other host receptors that act in concert with EPCR to mediate firm endothelial binding, as these adhesion traits may also influence microvascular pathology.

Although considerable work has been done on *var* gene expression in severe pediatric malaria,^18^,^107^,^108^,^113^,^116^ almost no information exists on DC8 or DC13 *var* gene expression in adult severe malaria. One of the aims of the India ICEMR is to investigate the expression of *var* genes in Indian adults. This question is also being evaluated as part of a collaborative effort between multiple independent ICEMRs using carefully clinically defined cohorts, in which patients in India have undergone MRI, fundoscopic examinations and have been evaluated for endothelial responsiveness to TNF. By having a precise comparison between MRI and fundoscopic findings, PfEMP1 expression, and host endothelial phenotypes, it may be possible to distinguish if host or parasite factors contribute to different pathological manifestations.

## Severe Vivax Malaria in Children and Adults

The other major *Plasmodium* species infecting humans is *P. vivax*. Although *P. vivax* infections are rare in most of Africa because of the high percentage of the human population with the Duffy blood group antigen–negative phenotype that is highly resistant to RBC invasion,^117^,^118^ it is estimated that over 2.5 billion people are at risk of *P. vivax* transmission. Approximately 91% of the populations at risk of transmission are in central and southeast Asia.^117^ Furthermore, in Brazil, *P. falciparum* cases are declining, and *P. vivax* has become the dominant parasite species in many endemic areas.^119^

Historically, *P. vivax* has been considered a relatively benign parasite, but recently there has been a renewed appreciation that it carries a significant morbidity and mortality burden in endemic regions.^21^,^120^,^121^ Furthermore, a 5–15% mortality rate was reported in the early neurosyphilis therapies of patients with *P. vivax*.^120^ Part of the explanation for the “benign” reputation, despite the evidence for mortality, is that vivax parasites are highly restricted to reticulocytes and therefore cannot achieve the same high parasite biomass as *P. falciparum*.^4^ A second difference is that *P. vivax* possesses relatively poor IE adhesive capacity compared with *P. falciparum*.^122^ Major questions for vivax pathogenesis include how does a parasite that is limited to lower grade parasitemias cause severe malaria? And is severe disease a consequence of vivax infection alone, the relapsing nature of *P. vivax*, or do other comorbidities influence disease severity? Within the ICEMRs, work is being done to better understand the prevalence and severity of *P. vivax* infections in Latin America and Asia and to characterize factors that may contribute to disease severity.

Clinically, vivax infections are associated with a chronic debilitating febrile illness that can be accompanied by chills, vomiting, malaise, and headache.^21^ On a per parasite basis, *P. vivax* is highly potent at inducing pro-inflammatory cytokines, such as TNF^21^,^120^,^121^,^123^,^124^ and has a much lower pyrogenic threshold than *P. falciparum* (180 vivax parasites/μL compared with 1,000 falciparum parasites/μL).^124^,^125^ The most frequent severe complications of vivax infection are severe anemia and acute respiratory distress.^121^ Cerebral malaria is a rare complication of *P. vivax* mono-infection, although it has been reported in India.^126^ In general, even less is known about the pathogenetic mechanisms in vivax malaria than *P. falciparum*, and it is not known if *P. vivax* CM cases reflect a particular strain of *P. vivax*, and/or a region-specific host susceptibility.

### Parasite adhesion and severe vivax malaria.

Unlike *P. falciparum*, *P. vivax* IEs become more deformable as they mature,^127^ and all parasite stages are visible in peripheral blood smears.^21^ However, late-stage schizont forms are underrepresented in peripheral blood,^119^ suggesting sequestration may occur. The lack of a continuous culture system has hindered research into *P. vivax* cytoadhesion, but the mechanism is distinct from *P. falciparum* because *P. vivax* IEs lack knob-like protrusions and do not encode *var* genes.^22^ Ex vivo studies have shown that *P. vivax* IEs adhere to placental cryosections as well as human lung—albeit at 10–15 times lower binding levels than *P. falciparum*.^128^ A strong candidate for *P. vivax* cytoadhesion and rosetting functions is a family of variant sub-telomeric genes named *vir*.^129^ On the basis of the sequence analysis, VIR proteins are classified into different groups, which have been found to have different subcellular localizations and functions.^130^ To study the cellular trafficking and adhesive functions of VIR proteins, they have been transfected into a poorly cytoadhesive *P. falciparum* strain (3D7), permitting gain of function studies. Two of three transfected VIR proteins were transported to the IE surface and one conferred ICAM1 binding activity.^131^ Whether cytoadhesion has a role in organ-specific disease complications is currently being investigated. There are few autopsy findings from polymerase chain reaction–confirmed *P. vivax* mono-infections. In one postmortem series from Brazil, ARDS and pulmonary edema was associated with accumulation of neutrophils in the interalveolar space, and scattered *P. vivax* IEs were present inside the pulmonary capillaries.^132^ A single autopsy performed in India showed monocyte infiltrates in alveolar capillaries.^133^ It has been postulated that *P. vivax* sequestration in pulmonary microvessels may trigger the inflammatory influx,^134^ but more work is needed to prove this hypothesis.

### Parasite invasion pathways and vivax malaria severity.

In contrast to the deadly *P. falciparum*, which is able to invade RBCs of all age, it has been suggested that the lack of fatalities from *P. vivax* malaria is related to its unique restriction to invasion and growth in reticulocytes. The Duffy blood group antigen on RBCs has a key role in invasion.^118^ This protein is recognized by the *P. vivax* Duffy binding protein (DBP),^135^ a leading vivax vaccine candidate. Although the identification of Duffy-dependent and Duffy-independent strains in Madagascar^136^ indicates that *P. vivax* can use alternative invasion pathways, it is unknown how extensively Duffy-independent strains are distributed throughout the world. In addition, a single amino acid polymorphism in the Duffy antigen Fy(a)/Fy(b) affects *P. vivax* invasion efficiency and the risk of clinical vivax in Brazil,^137^ but the effect of this polymorphism has not been examined in other parts of the world.

Despite the strong preference of *P. vivax* for reticulocytes, there is still a relatively poor understanding of why *P. vivax* is unable to invade normocytes or of the potential role of alternative invasion pathways in disease severity. A reticulocyte-binding protein complex was identified (PvRBP-1 and PvRBP-2), which plays a key role in reticulocyte binding and invasion.^138^ A related protein family was subsequently discovered in *P. falciparum* and named reticulocyte homology or RBL proteins. *Plasmodium vivax* genome sequences indicate the presence of numerous RBL paralogs,^22^ and intriguingly an additional DBP paralog,^139^ which might contribute to different modes of invasion, immune evasion, and pathogenesis. Within the India ICEMR, *P. vivax* in vitro invasion assays are being conducted to characterize the role of invasion pathways in disease severity.

## Cross-ICEMR Comparison of Research Activities Related to Severe Malaria

The ICEMR program covers a wide range of malaria transmission intensities for *P. falciparum* and *P. vivax*. Within the ICEMR program, nine ICEMRs based in south Asia, India, east and southern Africa, Amazonia, and southwest Pacific are collecting descriptive data on the characteristics and outcomes of patients admitted with severe malaria (Table 2). This broad approach can provide a better understanding of the relationship between severe malaria outcomes across the endemicity spectrum and may lend itself to meta-analysis to understand risk factors for incidence of severe disease. In addition, individual ICEMRs are investigating the role of prompt and effective therapies on minimizing severe malaria outcomes in African children and assessing the clinical profile and their association with the parasite and host immunological status and the role of nutritional factors and helminth coinfections in complicated malaria cases in Colombia (Table 2).

## Conclusions

Although the pathophysiology of CM is complex, pediatric autopsy studies have demonstrated two major patterns: cerebral microvessels with sequestered IEs alone (CM1) and cerebral microvessels with IE sequestration plus evidence of endothelial dysfunction and activation of coagulation (CM2). Ring hemorrhages and cerebral thrombosis are also described in a proportion of adult cases, but whether there is an equivalent CM1/CM2 dichotomy in adults is unclear. Neuroimaging studies have highlighted an important role for brain swelling in pediatric CM, which is less commonly observed in adult CM. A recent focus has been the microvascular interactions between *P. falciparum* IEs and cerebral endothelial cells, and how these binding interactions may contribute to disease presentation. Furthermore, because of the inaccessibility of cerebral microvessels, dermal biopsies provide a noninvasive approach to profile the endothelial reactivity of patients with severe or non-severe malaria complications. It has been postulated that EPCR-binding parasites associated with severe pediatric malaria may impair the protein C pathway in cerebral microvessels and thereby directly contribute to coagulopathy and endothelial barrier disruption. However, further work is needed to understand to what extent parasite adhesion or endothelial phenotypes may contribute to the pathophysiological differences between CM1, CM2, and adult CM.

By comparison to *P. falciparum*, the lower lethality of *P. vivax* may relate to invasion and growth in reticulocytes and lower cytoadhesive properties. Nevertheless, despite its benign reputation, there has been a surge in reports on severe vivax malaria and a growing appreciation that *P. vivax* is not harmless. Recent studies in Peru suggest that severe vivax can occur in monoendemic malaria regions.^140^ Although highly restricted to reticuloctyes, genome projects have revealed a large expansion of invasion ligand gene families in *P. vivax*. Thus, it will be important to investigate if invasion pathways influence vivax disease severity. Within the ICEMR program, current research efforts are focused on understanding disease mechanisms, as an important prerequisite to developing new tools to diagnose and treat severe malaria.

## Figures and Tables

**Table 1:** Histological characteristics of pediatric and adult CM

Characteristic	Pediatric		Adult
	CM1	CM2	CM
Intravascular pathology			
Infected erythrocytes in microvessels of gray and white matter	Yes^15^,^16^,^33^	Yes^15^,^16^,^33^	Yes^34^,^35^
RHs	No^15^,^16^,^33^	Yes^15^,^16^,^33^	Yes, subset^7^,^17^,^30^,^36^–^38^
Increased BBB permeability to plasma factors associated with RH	NA^15^	Yes^15^	Yes^13^
Increased BBB permeability to plasma factors associated with sequestered IEs	Yes^15^	Yes^15^	Yes^13^,^39^
Microvascular thrombosis associated with necrosis of endothelial lining and perivascular hemorrhages	No^15^	Yes^15^	Limited
Fibrin thrombi	No^15^,^16^,^33^	Yes^15^,^16^,^33^	Rare^30^,^31^,^37^
Pigment-containing monocytes	No^15^,^16^,^33^	Yes^15^,^16^,^33^	Yes^37^,^40^
Perivascular pathology			
Reactive astrocytes	Yes^15^	Yes^15^	Yes^40^
Durck's granuloma (reactive microglia, astrocytes, and lymphocytes)	No^15^	Extremely rare^33^	Occasional^41^
Axonal injury associated with RH or vascular thrombosis	NA	Yes^15^	Yes^40^
Myelin loss associated with RH	NA	Yes^15^	Yes^40^
Diffuse myelin damage associated with sequestered IEs	Limited^15^	Yes^15^	?
Axonal injury associated with sequestered IEs	Yes^15^	Yes^15^	?

BBB = blood–brain barrier; CM = cerebral malaria; IEs = infected erythrocytes; NA = not applicable; RHs = ring hemorrhages; ? = not reported.

**Table 2:** ICEMR activities related to severe malaria

ICEMR	Research activities related to pathogenesis of malaria
Southeast Asia	Collecting descriptive data on malaria patients attending local hospitals at sentinel sites, including data on disease manifestation
South Asia (India)	Investigating the molecular and cellular basis of severe *Plasmodium falciparum* and severe *Plasmodium vivax* infections in hospital patients recruited at multiple locations in India
India	Assessing the role of interindividual variations in endothelial responsiveness to TNF in the development of cerebral malaria
	Investigating the pathology of cerebral malaria in India patients using novel MRI techniques
	Collaborating with the southern Africa ICEMR (Malawi) on MRI findings in adults and children with severe malaria
East Africa (Uganda)	Investigating the role of prompt and effective therapy for minimizing the risk of severe malaria in cohorts of children living in high-endemic settings
	Collecting descriptive data on characteristics and outcomes of children admitted with severe malaria at six public hospitals in Uganda
Southern African (Zambia/Zimbabwe)	Collecting descriptive data on clinical diagnoses for persons seeking care at rural health centers
Southern Africa (Malawi)	Collecting hospital-based data on febrile illnesses (malarial and non-malarial)
	Collaborating with south Asia ICEMR (India) on MRI findings in adults and child
Amazonia	Observational, hospital-based observations of severe *P. vivax* malaria; 16S rRNA molecular and blood culture analysis of severe malaria cases
Latin America (outside Amazonia)	Clinical profile of malaria in different epidemiological settings in Colombia, and their association with parasite and host immunological status
	Determine the effects of immune status, nutritional factors, and helminth coinfection on complicated malaria cases in Colombia
Southwest Pacific	Collecting data on childhood severe malaria admissions to major hospital serving Madang Province

ICEMR = International Centers of Excellence for Malaria Research; MRI = magnetic resonance imaging; TNF = tumor necrosis factor.
